# Three-dimensional geometric morphometric sex determination of the whole and modeled fragmentary human pubic bone

**DOI:** 10.1371/journal.pone.0265754

**Published:** 2022-04-06

**Authors:** Katherine Baca, Brandon Bridge, Meradeth Snow

**Affiliations:** 1 Department of Anthropology, University of Montana, Missoula, Montana, United States of America; 2 Department of Economics, University of Montana, Missoula, Montana, United States of America; The Cyprus Institute, CYPRUS

## Abstract

Sex determination of the human pelvis has traditionally been done through visual analyses of morphoscopic traits and there are limited metric methods available to forensic anthropologists to add metric credibility to these analyses. The goal of this research was to create an improved metric method using three-dimensional geometric morphometrics to determine sex from both whole and modeled fragmented human pubic bones. The sample consisted of n = 378 pubic bones from the University of New Mexico’s Maxwell Museum Documented Skeletal Collection and eight landmarks were collected from each bone. Statistical analyses and machine learning algorithms were used to predict the accuracy of the method’s ability to classify a bone as male or female on both whole and simulated fragmented remains; this included tests run on each possible landmark combination of three or more landmarks to simulate fragmented bones (218 combinations). The results of the whole bone analysis resulted in 95.35% testing accuracy. The results of the modeled fragmentary analysis consisted of 164 combinations which exhibit a 90% or higher accuracy in sex prediction; and twelve combinations which exhibit 96% or higher accuracy in sex prediction. In particular, two landmarks clustered around the ventral arc of the pubic bone performed the best, indicating this is the most sexually dimorphic portion of the bone. These results indicate that three-dimensional geometric morphometrics is a valid method to be applied to sex determination in forensic anthropology.

## Introduction

Sex determination using the pelvis has traditionally been done using a series of morphoscopic traits, including the overall shape of the pelvic inlet, the ventral arc, the ischiopubic ramus, subpubic concavity, and the greater sciatic notch [1,2]. It is well accepted that the pelvis is the best element to use to determine sex when available [3–5]. An experienced biological anthropologist can generally estimate sex from a visual analysis of the pelvis correctly approximately 90–95% of the time [1,3,4,6,7]. The pelvis exhibits a series of sexually dimorphic differences as an individual matures, including a wider pelvic inlet and sub-pubic arch for females, and a narrower greater sciatic notch and pubic bone for males, most of which are due to the physiological ability of females to give birth [1,3,6–8]. Few accepted metric analyses of these shape differences in the pelvis exist, but metric analysis is becoming more important as it becomes more common for forensic anthropologists to testify as experts within the court system [3,9].

Geometric morphometrics is a metric analysis of shape and can be performed in both two-dimensional and three-dimensional planes [10]. This research applies 3-D geometric morphometrics to the problem of metrically determining sex from the human pelvis. This method is advantageous because it offers more metric information about the specimen than traditional visual or interlandmark distance measurements do. Traditional visual analyses rely on the expertise of the forensic anthropologist to judge the size and shape of the bone by simply examining it. Completing a statistical analysis on multiple points in space (landmarks on bone) offers measurements between the x, y, and z coordinates of each landmark and a much more complete and exact analysis of the bone as a whole; it also offers the chance for digital manipulation of the data which can produce more complex and insightful results than traditional methods [10]. A 2011 study combined both visual analyses and basic 3-D analyses from CT scans of the pelvis to estimate sex [11]. This study resulted in a 100% accuracy rate, exhibiting that it will always be important to understand and integrate existing techniques with new methods and data [11]. Other studies utilizing CT scans of the pelvis have also exhibited differences between males and females [12–14]. Geometric morphometric analyses are also advantageous because they consist of non-destructive techniques. Metric analyses are also more objective and generally require less training and experience than visual analysis techniques [15]. Geometric morphometrics is commonly utilized in biological studies and has more recently become a more commonly utilized method of analysis in anthropological studies as well [16–18]. Existing research in biological anthropology mainly utilizes the method for cranial ancestry estimation and age-at-death estimation, and there are few metric methods available for sex determination from the pelvis [19,20].

Geometric morphometrics can be used to analyze 2-D data as well as 3-D; multiple studies have used 2-D geometric morphometric analysis to exhibit shape differences between male and female pelves [21–24]. The DSP2 method is one of the few large-scale metric methods of sex determination using the pelvis and has been shown to be quite accurate [25,26]. This method utilizes interlandmark distance measurements across the entire os coxa; in order to obtain reliable results using this method the individual in question must present a nearly complete os coxa, which can be problematic in the field. Another study, similar to the present research, utilizes 3-D geometric morphometrics to analyze landmarks over complete os coxae [3]. It is, however, rare to recover complete sets of skeletal remains in most forensic and archaeological scenarios [3]. Often, remains have been buried or exposed to the elements for some time before recovery, which results in broken, partially disintegrated, and/or incomplete bones available for analysis. Due to this, it is necessary to develop methods of sex estimation that can be used on smaller portions of the pelvis and fragmentary remains.

It is important to develop updated methods using a contemporary collection in order to ensure the method’s accuracy when applied to modern forensic cases [27]. This helps to avoid biases that may be introduced by secular change in a population. Anatomically modern *Homo sapiens* have existed for approximately 200,000+ years and the modern human skeleton has changed throughout this time due to the environment and random genetic drift, a concept referred to as secular change [28,29]. Due to this morphological change over time, it is important to develop new methods and research on contemporary collections of skeletal remains [30]. A recent study showed that secular change can happen relatively quickly, and can affect traits on the pelvis that are regularly used in forensic anthropology [28]. Samples from the Hamaan-Todd Osteological Collection and the William M. Bass Donated Skeletal Collection were compared to denote any changes in the ischiopubic ramus, the subpubic angle, and the ventral arc of the pelvis [28]. The Hamaan-Todd Collection includes individuals that were born between the mid-nineteenth century and the early twentieth century, while the William M. Bass Collection includes individuals born since 1940 [28]. The results showed significant shape differences in all three traits for females, and the ventral arc for males [28]. The sex determination methods which utilize these traits still offer high accuracy determinations for both populations, but with such significant shape changes occurring over time, it is unknown how long changes will continue to not affect the methods used [28]. This study demonstrates the need for continued contemporary skeletal collections.

The goal of the present research is to provide a refined method using a 3D Microscribe Digitizer to assess sex from the pubic bone in both a whole and fragmentary state. This research refines similar methods used in previous studies which utilized a 3D Microscribe Digitizer to estimate sex from landmarks located across the entire os coxa [3]. This research also noted patterns in morphological change related to age, ancestry, and parity in females, all of which likely play a role in pelvis morphology, but to what extent is unclear. The first hypothesis guiding this research is that if there are significant differences in the shape of male versus female pubic bones, then a geometric morphometric analysis will be able to distinguish between the two sexes and accurately determine whether an unknown bone belonged to a male or female. The method will be considered accurate if the predictions are consistent with or better than current morphoscopic techniques that can determine sex correctly approximately 90–95% of the time [1,3,15]. If the shape differences between males and females are not significant, then the geometric morphometric analysis will be no better than chance, with prediction rates at approximately 50%. The second hypothesis is that if significant shape differences are detectable using this method, then the differences will also be detectable when modeling fragmentary pubic bones and that certain landmarks will be more effective than others in establishing a sex determination.

## Materials/Methods

**Fig 1:**
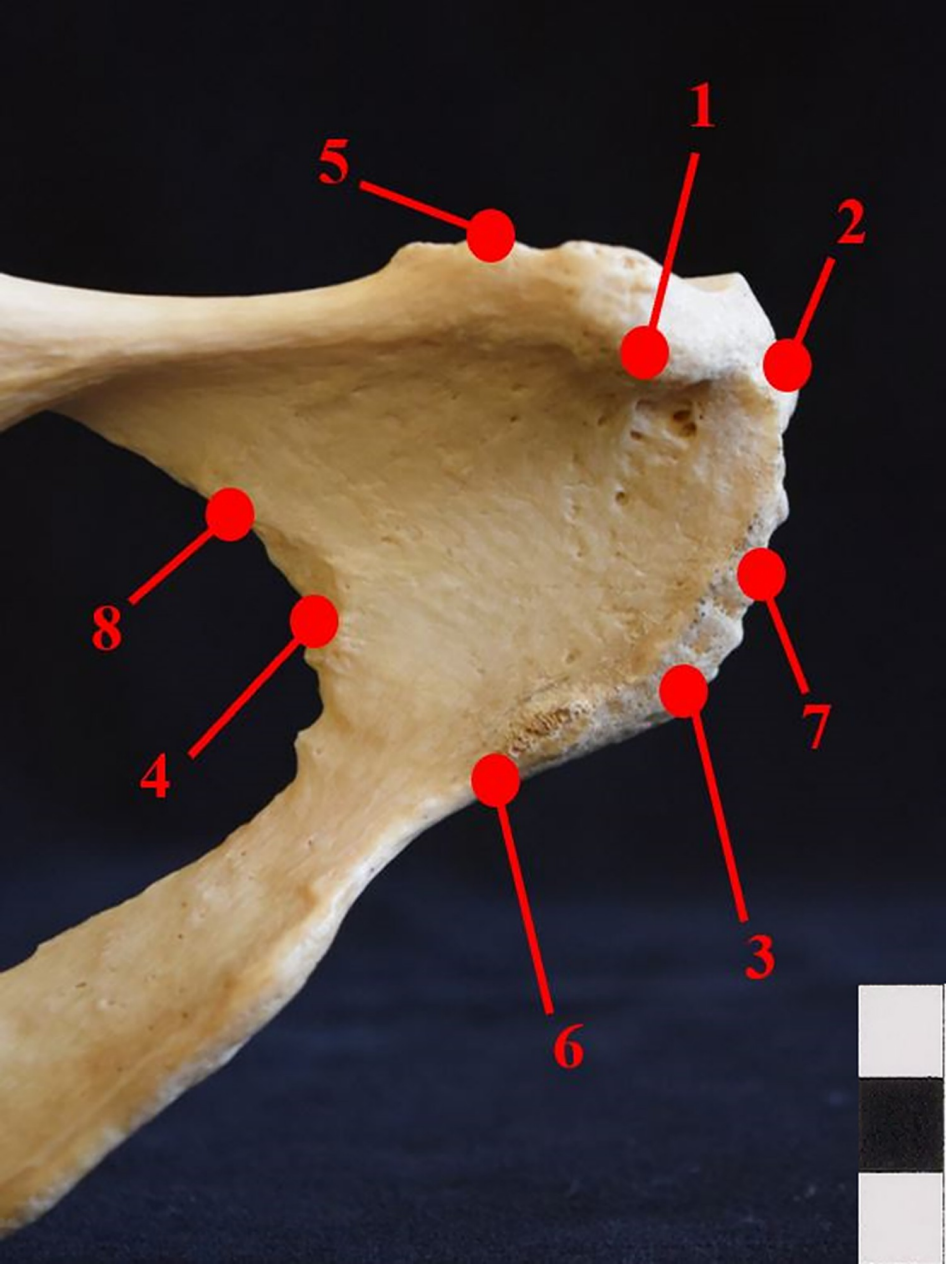
Location of landmarks. Anterior surface of the right pubic bone displaying approximate locations of the eight landmarks recorded on each bone; scale bar is in 1 cm blocks.

In order to perform a 3-D geometric morphometric analysis of the pubic bone, a Microscribe G2X digitizer was utilized to collect a set of landmarks from each specimen. Eight landmarks were collected on each specimen when available, only specimens that were missing three or fewer landmarks were utilized. See Table 1 for the description of the landmark locations and Fig 1 for an image of the approximate location of each landmark.

**Table 1:** Description of the location and landmark type of the eight landmarks recorded on each bone.

Number	Landmark	Description
1	Pubic Tubercle	Most prominent point of the pubic tubercle
2	Superior Pubic Symphysis	The most superior point of the pubic symphysis
3	Inferior Pubic Symphysis	The most inferior point of the pubic symphysis
4	Lateral Border	Point on the lateral border of the pubic body which would create the maximum breadth of the obturator foramen
5, 6	Pubic Body Height	The superior (5) and inferior (6) points which create the maximum height of the pubic body
7, 8	Pubic Body Width	The medial (7) and lateral (8) points which create the maximum width of the pubic body

The landmarks were chosen by the author based on previous research and a pilot project which aided in narrowing down landmarks that were easily identified and replicable [3]. When collecting data on landmark points, it is important to distinguish which type of landmark it is. Landmarks can be categorized as type I, II, or III [10,31,32]. Type I landmarks are considered the easiest to find and consist of one single point on the bone where tissue transitions, such as an intersection of sutures [3,33,34]. Type II landmarks are considered the points of maximum curvature or greatest muscle attachment, an example would be the euryon on the cranium [33,34]. Type III landmarks are the most extreme points of a structure overall, sometimes labelled as the “posteriormost” or “anteriormost” points [3,33].

213 individuals were utilized from the University of New Mexico’s Maxwell Documented Skeletal Collection, which resulted in 378 total pubic bones. The Maxwell Documented Collection consists only of donated individuals who have passed away in the past 50 years, making it one of the largest modern collections in the US. The collection houses over 300 individuals, 60% of which are male, the majority of adults are aged 51–75, and the sample as a whole is self-identified as 80% White (see Table 2 for demographic information) [35]. All 213 individuals utilized were 18 or older to avoid the indeterminate morphology present in juvenile pelves, and when available, both right and left pubic bones were recorded.

**Table 2:** Demographic information.

	Number of Individuals	Parous	Non-Parous	Age Group 1: 18–35	Age Group 2: 36–50	Age Group 3: 51–60	Age Group 4: 60+	White Ancestry	Hispanic Ancestry	Black Ancestry
Male	133	N/A	N/A	17	16	31	69	112	5	4
Female	80	34	10	2	6	8	64	78	4	1
Total Number of Individuals	213	34	10	19	22	39	133	190	9	5

Demographic information including the number of individuals, parous and non-parous females, age groups, and self-reported ancestry of the 213 individuals included in the sample, note that parity and ancestry information was not available for all individuals.

The statistical analysis began with transferring the raw data from the spreadsheets into Python 3 [36]. Before the data is manipulated in any way, it contains information on size, position, and orientation; all of which must be removed in order to analyze shape alone [32,37]. A Generalized Procrustes Analysis was run to transform the data so that it is scaled and rotated to the same plane, leaving only the shape information [10]. The next step was a Principal Components Analysis, or PCA. A PCA is a way to represent the variation that is present within the sample, and the goal of a PCA is to determine which variable introduces the most amount of variation [38]. After completing the PCA, outliers were removed from the sample for all subsequent analyses; this resulted in an overall sample size of 374 pubic bones.

Lastly, a Discriminant Function analysis was run through Python to determine the predictive power of the sample based on the two groupings of males and females. The analysis randomly breaks the data set into two sets, placing 70% of the data in the first and the remaining 30% of the data into the second. The first set is used as a training set to teach the software the difference between the two classifying groups (male and female). The second set is used as a test to predict how well the machine learned to distinguish between the two groups. The end result is the predictive power of the method’s ability to determine whether an unknown bone originated from a male or a female individual. The first discriminant function was run on the entire sample to establish a baseline for how well the method worked when presented with whole, non-fragmented bones.

Next, to model fragmentary scenarios, every possible combination of three landmarks or more was run through a discriminant function test. The missing landmarks simulate missing portions of bone. The first combination consisted of landmarks one, two, and three; the next one, two, and four; and so on up to all combinations of seven of the eight landmarks. This provided 218 possible combinations, or simulated fragmented bones, each of which, provide a Discriminant Function Analysis of each specific combination’s predictive power between the two classes of male and female.

In order to determine ease of reproducibility, the author resampled 50 individuals from the previously sampled 213. The author collected data for a second time on each of the 50 individuals, N = 100 pubic bones. One of the advantages of geometric morphometrics is that the data can be collected on any plane and rescaled to perform analysis; because of this, one of the clearest ways to assess replicability was to simply run the entire statistical process again using only the resampled data and compare these results to the results of the larger sample. This analysis was performed exactly as described above, using Python software.

To test the presence of interobserver error, the author collected all eight landmarks from a single bone, once a day, six days in a row, to determine whether or not the landmarks were consistently recorded in the same location. The six instances of data collection were added to the original raw data collection of 378 individual bones. This data set was then analyzed through a GPA and PCA to visualize whether or not the data recording was precise enough for each of the five instances of recorded data from the same individual to plot close to each other.

**Fig 2:**
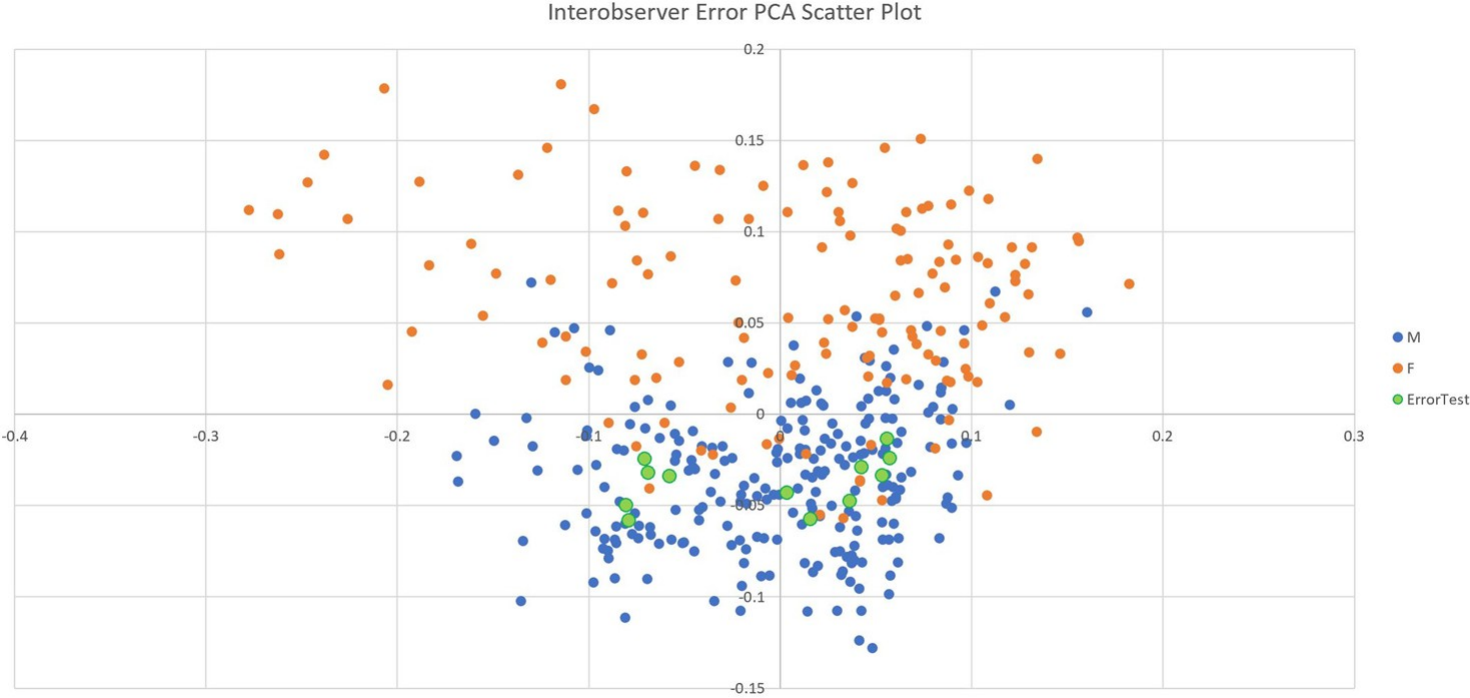
Interobserver error PCA scatter plot. This figure shows the original PCA scatter plot with the addition of the individual bone which was repeatedly digitized (the additional data points have been enlarged to aid in viewing).

Fig 2 displays the PCA scatter plot of the repeated individual added to the initial sample overall. The six instances of data collection from the same individual all fall squarely within the distribution of the rest of the data. All six instances are within .2 of the Euclidian distance of each other.

In order to check the robustness of the sample as a whole and to ensure that using both right and left pubic bones did not synthetically increase predictive power, a GPA, PCA and Discriminant Function Analysis was run on only the left-sided pubic bones in the sample. After removing all right-sided bones, the data set consisted of 198 individual left bones. Results similar to the results of the Discriminant Function Analysis of the sample as a whole (both right and left bones) will indicate that right and left symmetry between the bones of individuals is not biasing the sample towards a higher predictive power.

## Results

**Fig 3:**
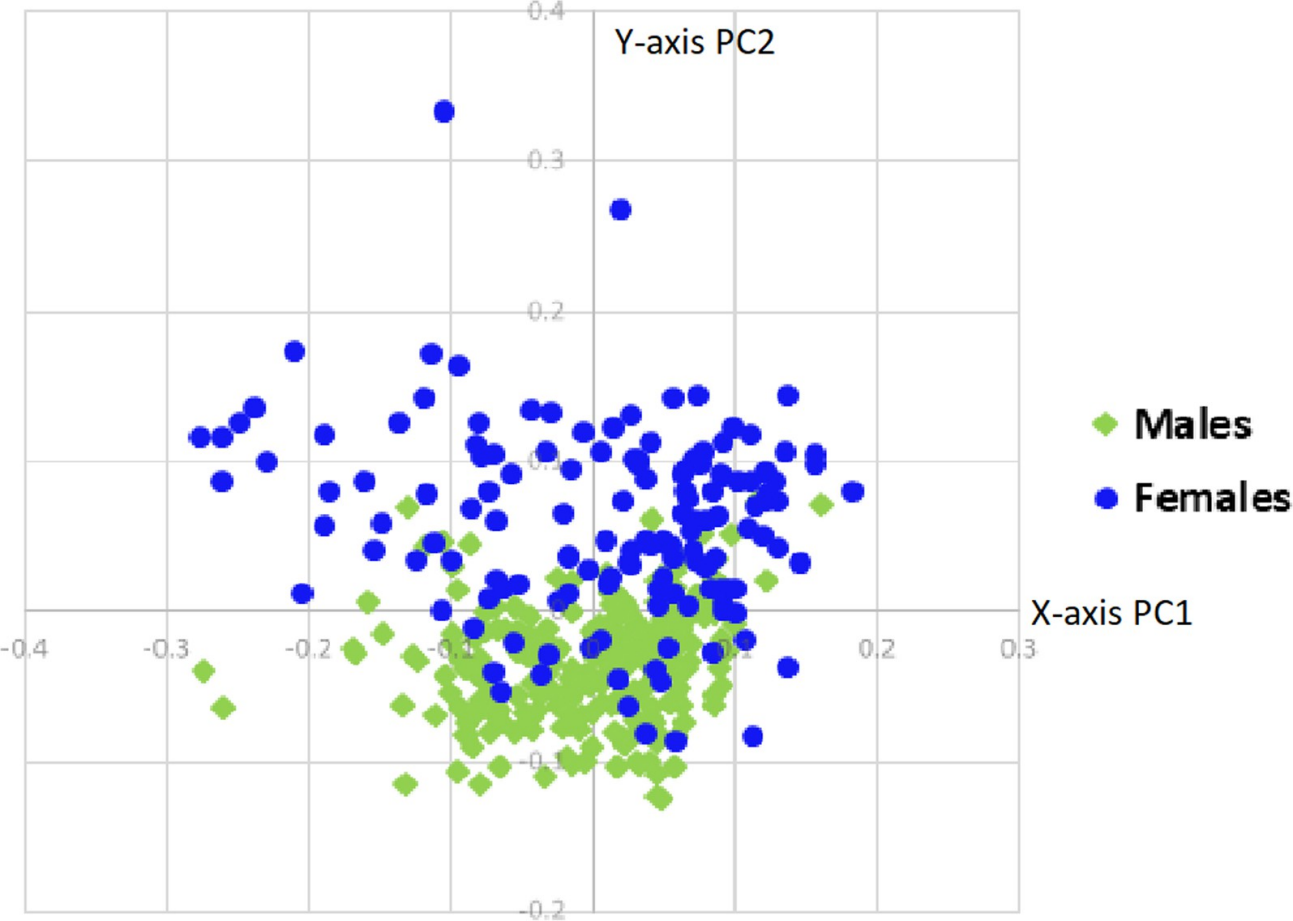
PCA scatter plot. PCA scatter plot displaying male and female groups.

**Fig 4:**
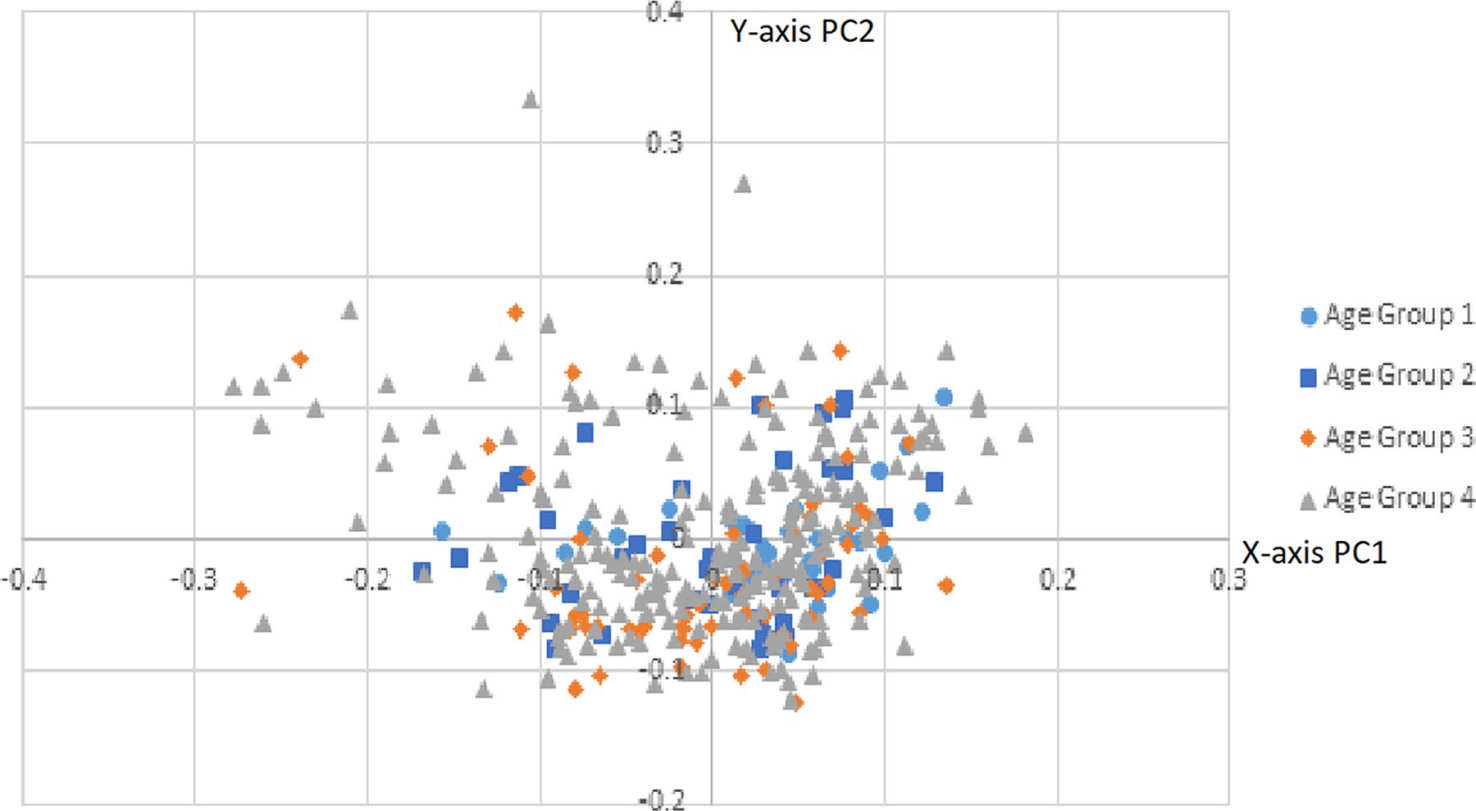
PCA age scatter plot. PCA scatter plot displaying age groups (males and females grouped together); group 1 (18–35), group 2 (36–50), group 3 (51–60), group 4 (61+).

**Fig 5:**
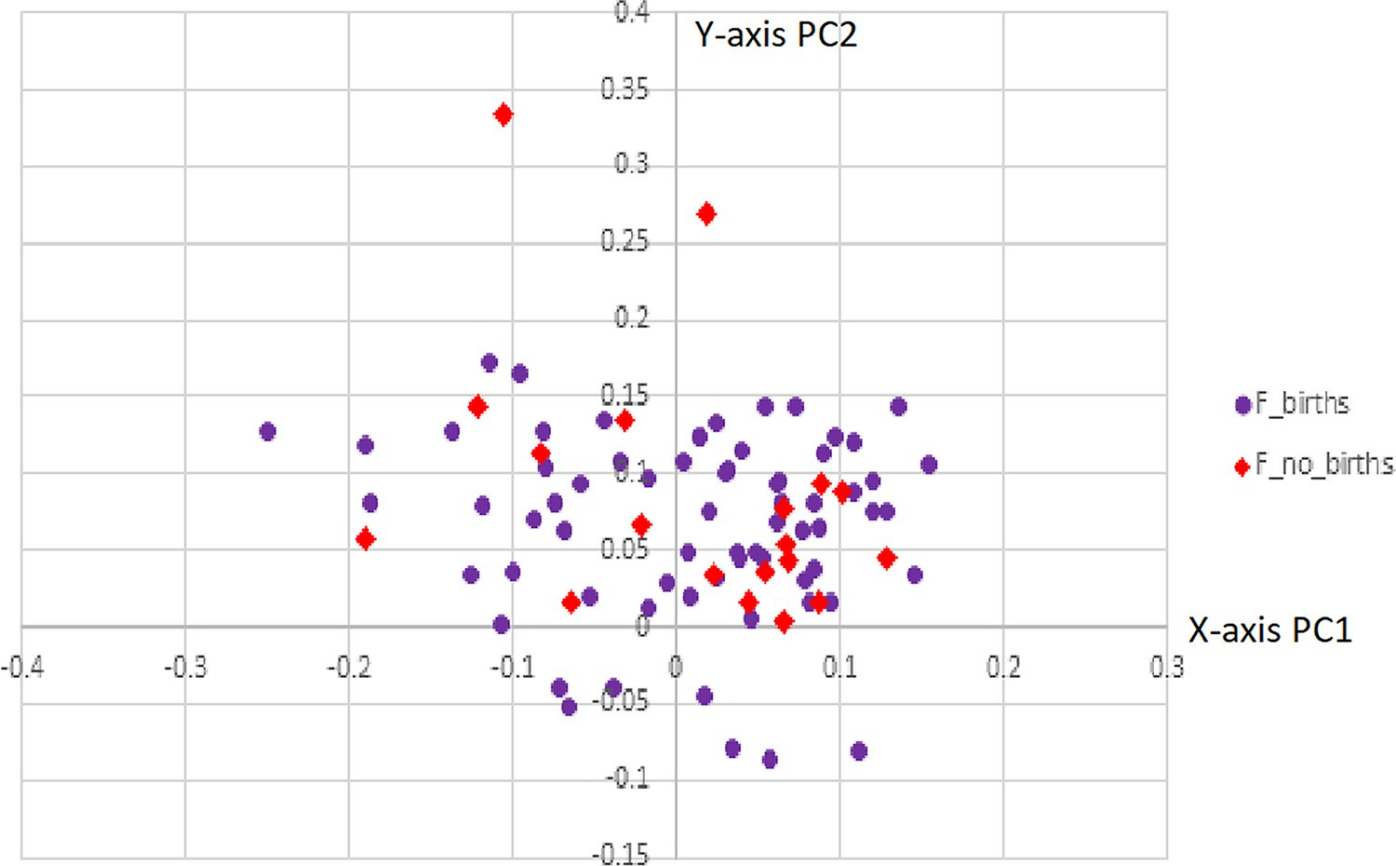
PCA parity scatter plot. PCA scatter plot displaying available data on parity of females (parous vs. non-parous).

The first two Principal Components (PCs) of the PCA test represented 43% of the total variance, which was close to, but not exceeding the ideal 50% for class separability. The resulting PCA scatter plot displaying PC one and two can be seen in Fig 3. The third PC represented 11.4% of the total variance, but was not presented here because it did not add clarity to the visualization of the data. The scatter plot was also coded to show males and females, age groupings, and parity of females (see Figs 3–5). While it does not in itself offer definitive class separability, it is clear from Fig 3 that the males and females do show some difference in how they cluster, and the male specimens cluster much more tightly than the females. It should be noted that the only African American female individual in the sample is represented by the two uppermost outliers of the scatter plot (right and left pubic bones). While a sample size of one is not nearly enough to confirm the necessity of an ancestry-specific method, this does indicate that future research should use larger and more diverse samples to determine whether or not ancestry is a defining variable.

By coloring the scatter plot based on age groupings, it is clear that as the sample groups get older, the variance becomes greater (Fig 4). The younger age groups (groups 1 and 2) cluster much tighter than groups 3 and 4. By coloring only the females with available data concerning their parity, it can be seen that parous females exhibit a slightly greater variance than non-parous females (Fig 5). Unfortunately, this data was not available for every female individual; however, this data indicates the need for future research, especially considering how little is known about the shape changes a female pelvis experiences after giving birth [39,40].

The first discriminant function test, which included all available landmarks for all 374 specimens resulted in 95.35% accuracy based on the training data, and 95.49% accuracy based on the testing data, P < .0001; with a Wilk’s lambda value of 0.2487 (see Table 3 for calculated significance checks). These results are on par with other sex determination methods on the pelvis, which as mentioned earlier range from approximately 90–95% [1,3,4,6,7].

**Table 3:** Test statistics.

Wilk’s Lambda	0.2487
Pillai’s Trace	.7709

Test statistics displaying significance of variables within the discriminant function. A Wilk’s Lambda close to zero indicates significant discrimination is present. A Pillai’s trace value close to 1 indicates the same and that the null hypothesis can be rejected.

The larger discriminant function loop which tested all possible combinations of three landmarks or more offered results for 218 different combinations. The least effective combination consisted of landmarks 1, 2, and 5 and resulted in a training score of 84.88% and a testing score of 79.27% accuracy. The 12 most effective combinations of fragments all resulted in testing scores of 96% and higher; see Table 4 for the landmarks included in each of these combinations. It should also be noted that 164 of the 218 (75%) possible combinations resulted in testing scores of 90% or higher. Results for all 218 landmark combinations can be viewed in the supplemental information.

**Table 4:** The twelve landmark combinations which resulted in the highest testing scores, showing their corresponding training scores and which landmarks were included in each combination.

Combination Number	Training Score	Testing Score	Landmarks Included
40	93.79%	96.39%	3, 4, 8
116	94.57%	96.39%	3, 4, 6, 8
121	94.96%	96.39%	3, 6, 7, 8
128	94.96%	96.39%	1, 2, 3, 4, 6
139	95.34%	96.39%	1, 2, 4, 5, 8
155	94.18%	96.39%	1, 3, 5, 7, 8
167	94.6%	96.39%	2, 3, 4, 7, 8
170	94.96%	96.39%	2, 3, 5, 7, 8
173	95.73%	96.39%	2, 4, 5, 6, 8
106	93.02%	97.29%	2, 4, 6, 8
115	94.96%	97.29%	3, 4, 6, 7
132	94.18%	97.29%	1, 3, 5, 7

The second data set which consisted of 50 individuals, n = 100 pubic bones, to test replicability of the method, resulted in a training set accuracy of 96.5%, and a testing set accuracy of 93.8%. This demonstrates a successful second use of the method on a separate, albeit smaller, data set.

The Discriminant Function Analysis of only the left bones resulted in a training accuracy score of 96.09% and a testing accuracy score of 94.54%. A result this high indicates that the data is robust and that right and left symmetry is not biasing the sample.

## Discussion

The results reported here clearly indicate that this 3-D geometric morphometric method of sex determination has the potential to increase the accuracy and credibility of sex estimations on both whole and fragmented human pubic bones. The sample overall, using all eight landmarks, predicted sex correctly 95.49% of the time. This result alone indicates that this method is effective and that 3-D geometric morphometrics can be used to distinguish between the sexes on smaller portions of the pelvis. Experienced forensic anthropologists using morphoscopic methods can accurately determine sex approximately 90–95% of the time, depending on the state of the bones and the experience of the user [1,3,15]. Similar studies, which collected landmarks across the entire os coxa reported similarly high results, so it is promising to see that the method can be narrowed down to a smaller portion of bone and still retain high accuracy [3,15].

The results of the fragmentary modeling analysis are also quite promising; three-quarters (75%) of all possible combinations of these landmarks resulted in an accuracy rate of 90% or higher, which is in the same range as traditionally used visual analysis methods [1,3,4,6,7]. This result indicates that this method could be used on many differently sized and broken fragments of pubic bone to gain an accurate estimation of sex. It was noted that the landmarks most commonly physically missing from the skeletal collection at UNM were landmarks 1, 2, and 5. To represent an actual fragmentary context, the fragment combination missing those landmarks (combination 180) resulted in a testing accuracy of 95.49%. This is just as high as the sample overall and displays that this method worked quite well in a “real” fragmentary context. Finally, twelve simulated fragments resulted in an accuracy result of 96%, which is slightly higher than the overall accuracy of the method when all eight landmarks are included in the discriminant function analysis.

**Fig 6:**
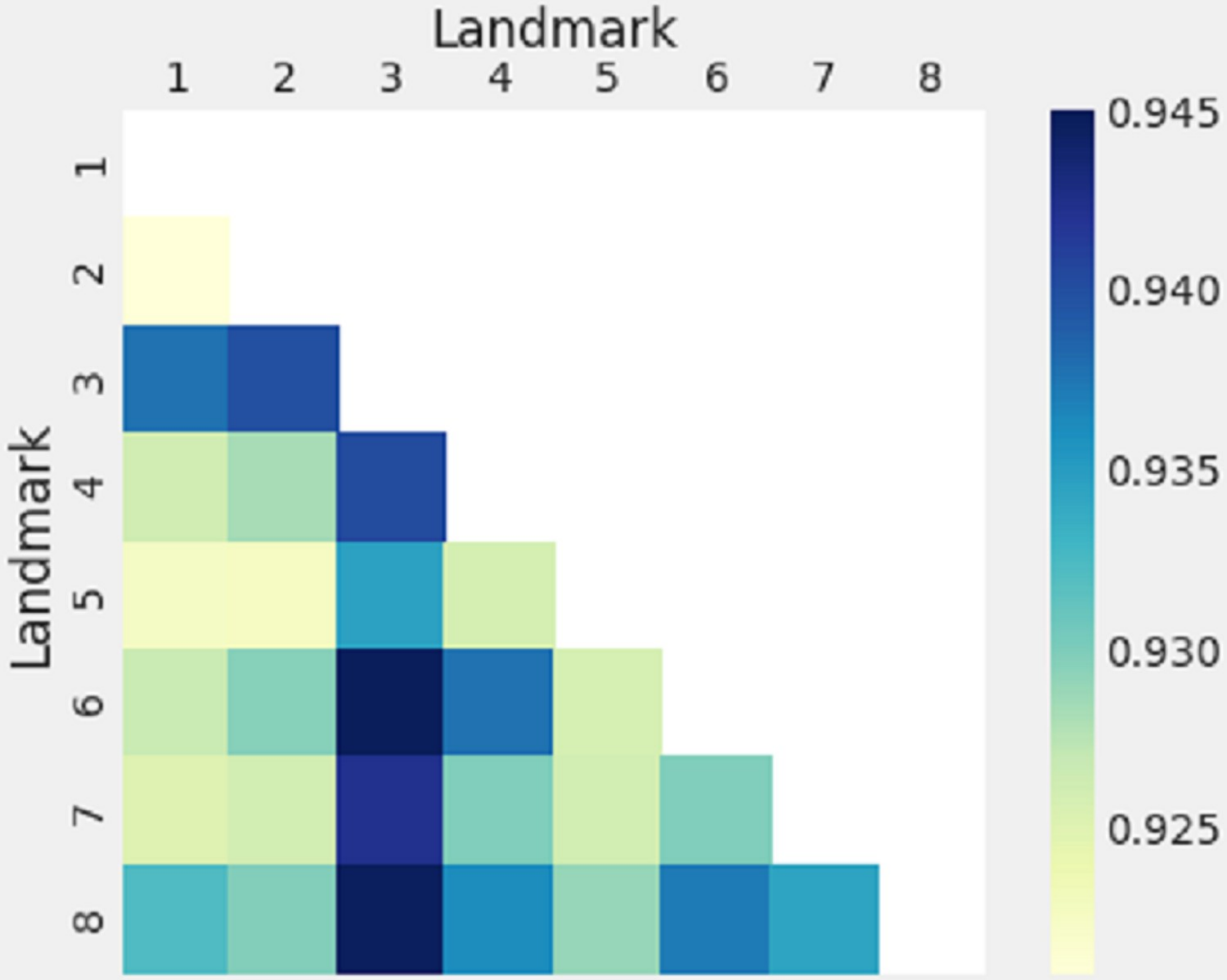
Landmark pair heatmap. Heatmap displaying the average predictive powers of combinations including each landmark pair; note that landmarks three and six make consistently more powerful combinations than other landmarks.

When considering the twelve combinations of landmarks that all resulted in 96% or higher accuracy, there are two specific results within that appear quite significant to the applicability of this method. First, combination 40 utilized only landmarks three, four, and eight, which is important because the method could still be effective when the entire antero-medial portion of the bone is missing. Landmark 3 appeared to be utilized in the majority of the most successful combinations, so to determine which landmarks exhibit the most predictive power, a heat map was created to visualize the more powerful landmarks. Fig 6 displays a heat map of the landmarks’ predictive powers. To create this map, the average predictive power of each combination containing landmarks one and two was calculated; the same for each combination containing landmark one and three, one and four, and so on with every two-landmark pair. The darker the color, the higher the average predictive power for combinations including that landmark pair. It is clear that combinations including landmarks three, six, and eight have the highest average predictive powers. The variance in the average of the predictive powers is not large simply due to the high accuracy of the data as a whole. Landmarks three and six are both located on the inferior portion of the bone, around the area of the ventral arc; the ventral arc is known to be one of the more reliable visual indications of male or female [7]. This is quite promising to the real-world applicability of this method because it indicates that very high results could still be obtained even if the forensic anthropologist is only presented with a small piece of the inferior pubic bone. At this point, the expert could perform both a visual analysis on the ventral arc, as well as a metric analysis using this method to make their overall sex determination.

Adding to the complicated nature of fragmentary remains is the knowledge that metric analyses are more trusted by court-systems and juries than are subjective morphoscopic analyses [9]. An expert should use multiple methods to validate their determinations, and a precise metric method to validate a sex determination could aid in establishing the credibility of the testimony. This method could also be useful in other contexts, such as mass graves, commingled and fragmentary remains, and bioarchaeological studies. In archaeological contexts when multiple individuals are identified, one of the first demographics determined is how many males and females are present; this method could easily offer a metric answer to this question. Another advantage of metric methods is that they allow for digital manipulation and analysis, which allows for the option of more in-depth results. Geometric morphometric analysis is also non-destructive and allows the observer to retain the original integrity of the sample. Employing metric methods also allows for the inclusion of interdepartmental collaboration among various subdisciplines of anthropology as well as mathematics and statistics [41]. As a field of study overall, these methods can continue improving metric analyses to aid in the forensic anthropologists’ biological profile determinations.

A limitation of this simulated fragmentary research is that not all of the high scoring landmark combinations reflect real possible fragmented bones. For example, combination 155 includes landmarks one, three, five, seven, and eight, and requires that essentially all sides of the bone are present, which is highly unlikely if the bone is fragmented. This is the case with most of the combinations which require more than four landmarks. Identifying combinations that only require three or four landmarks results in much more applicable results and again indicates that this is a promising method to continue developing. Combinations 40, 116, 115, and 132 (Table 4) are particularly applicable when it comes to simulating actual broken bone because each combination uses landmarks that are relatively close together (Fig 1). A second limitation is that this research was conducted using a sample of primarily white individuals, and further research into ancestry specific biases is needed.

It is important to recognize that “White” is an ambiguous term when it comes to determining ancestry or regional belonging; “White” can mean many things, and a White sample from New Mexico will likely be quite different from a White sample elsewhere. Although, it is at least a similar, overall characteristic that can be used to combine and contrast future population data. Ancestry classifications are also self-reported, which means they represent which group each individual identified with, and not necessarily where their genetic ancestors came from. In reality, white is a color and not an ancestry, meaning that the genetic ancestry of people who identify as “White” could vary drastically. It has also been shown that those who are multiracial, or identify with multiple ancestries or ethnicities, often change their identification over time, and usually choose to list a single ancestry rather than multiple ones [29]. Self-reported information on ancestry may not be the most reliable, however it does create a group that can still offer important information when compared and contrasted, so long as the general issues with the classification are taken into account.

It is imperative to develop methods which can be used on fragmentary remains, considering how often incomplete remains are recovered. This is true not only in forensic contexts, but mass graves, commingled remains, and bioarchaeological contexts as well. It is likely that in these scenarios not all elements would be complete, meaning that an accurate metric method on a small portion of bone would offer an advantage to the anthropologist [3]. This method has been narrowed from the entire os coxa, to just the pubic bone, and then further to fragmentary scenarios, ensuring that it is applicable and useful to actual recovered remains [3]. A forensic anthropologist should use multiple methods to support their determinations and there are very few metric options available for sex determination. This method offers a very accurate, metric determination which could be added to the anthropologist’s traditional analysis. It is also vital that this method was developed using a sample which only contains individuals that have died in the last 50 years because it is more applicable on modern populations and there is less concern of secular changes biasing the results when used on contemporary individuals.

Based on the promising results here, future research into this method is needed. The method should be applied to larger samples to continue validating it as an established method so that it can be used in legal contexts in the future. The specific accuracy of traditional morphoscopic sex estimation methods on the Maxwell collection has not been established at this time; future research into this could offer vital comparative information to aid in the development of this 3D method. This research should continue to develop the fragmentary application of the method as well; replication of this research is vital if it is to ever be applied to actual forensic casework. Any ancestry specific biases should also be further explored on larger samples to ensure the method can be used across populations. This method may also have great potential in exploring the shape changes in female pelves related to pregnancy and birth. The PCA indicated that the female specimens exhibited more variation overall than did male specimens—this may be due to the traumatic event of giving birth, however more research is needed to determine whether or not this is the case.

## Supporting information

S1 Table Modeled fragmentary combination dictionary. This table lists each modeled fragmentary combination and which landmarks were included in each combination [42].
(CSV)

S2 Table Modeled fragmentary combination results. This table lists each modeled fragmentary combination and its discriminant function testing accuracy result [42].
(CSV)
